# COVID-19 vaccination coverage in Egypt: a large-scale national survey – to help achieving vaccination target, March-May, 2022

**DOI:** 10.1186/s12889-023-15283-w

**Published:** 2023-02-27

**Authors:** Amr Kandeel, Ibrahim Eldeyahy, Hanaa Abu ElSood, Manal Fahim, Salma Afifi, Shaimaa Abu Kamar, Hala BahaaEldin, ElSabbah Ahmed, Amira Mohsen, Khaled Abdelghaffar

**Affiliations:** 1 Preventive Sector, Ministry of Health and Population, Cairo, Egypt; 2 Ministry of Health and Population Public Health Consultant, Cairo, Egypt; 3 Community Medicine Department, National Research Centre, Cairo, Egypt; 4 Minister of Health and Population, Cairo, Egypt

**Keywords:** COVID-19 vaccines, Cross-sectional survey, Vaccine coverage, Egypt

## Abstract

**Background:**

Only 57 countries have vaccinated 70% of their population against COVID-19, most of them in high-income countries, whereas almost one billion people in low-income countries remained unvaccinated. In March–May 2022, Egypt's Ministry of Health and Population (MoHP) conducted a nationwide community-based survey to determine COVID-19 vaccine coverage and people's perceptions of vaccination in order to improve COVID-19 vaccination uptake and confidence among Egyptians, as well as to prioritize interventions.

**Methods:**

A cross-sectional population-based household survey among Egyptians ≥ 18 years of age was implemented in two phases using a multistage random sampling technique in all of Egypt’s 27 governorates. A sample of 18,000 subjects divided into 450 clusters of 20 households each was calculated in proportion to each governorate and the main occupation of the population. Participants were interviewed using a semistructured questionnaire that included demographics, vaccination information from the vaccination card, history of COVID-19 infection, reasons for vaccine refusal among the unvaccinated, and vaccination experience among vaccinated subjects. Vaccination coverage rates were calculated by dividing numbers by the total number of participants. Bivariate and multivariate analyses were performed by comparing the vaccinated and unvaccinated to identify the risk factors for low vaccine uptake.

**Results:**

Overall 18,107 were interviewed, their mean age was 42 ± 16 years and 58.8% were females. Of them, 8,742 (48.3%) had COVID-19 vaccine and 8,020 (44.3%) were fully vaccinated. Factors associated with low vaccination uptake by multivariate analysis included: age groups (18–29 and 30–39) (ORs 2.0 (95% C.I. 1.8–2.2) and 1.3 (95% C.I.1.2–1.4), respectively), residences in urban or frontier governorates (ORs 1.6 (95% C.I. 1.5–1.8) and 1.2 (95% C.I. 1.1–1.4), respectively), housewives and self-employed people (ORs 1.3 (95% C.I. 1.2–1.4) and 1.2 (95% C.I. 1.1–1.4), respectively), married people (ORs 1.3 (95% C.I. 1.2–1.4), and primary and secondary educated (ORs 1.1 (95% C.I. 1.01–1.2) and 1.1(1.04–1.2) respectively). Vaccine hesitancy was due to fear of adverse events (17.5%), mistrust of vaccine (10.2%), concern over safety during pregnancy and lactation (6.9%), and chronic diseases (5.0%).

**Conclusions:**

Survey identified lower vaccination coverage in Egypt compared to the WHO 70% target. Communication programs targeting the groups with low vaccine uptake are needed to eliminate barriers related to vaccination convenience, side effects, and safety to effectively promote vaccine uptake. Findings from the survey could contribute significantly to vaccination promotion by guiding decision-making efforts on the risky groups and preventing vaccine hesitancy.

## Introduction

Safe vaccines developed in less than a year are considered an end-of-game tool for ending the COVID-19 pandemic [1]. The World Health Organization (WHO) Director-General noted that only 57 countries have vaccinated 70% of their population, most of them in high-income countries, whereas almost one billion people in low-income countries remained unvaccinated [2]. Low- and middle-income countries (LMICs) are still facing considerable obstacles in receiving, distributing, and accepting vaccination at the community level. WHO is targeting global vaccination coverage of 70% -as soon as possible- by ensuring fair and equitable access to vaccines to limit virus transmission, its devastating impacts, and opportunities for further mutations [3].

Even if the countries have sufficient COVID-19 vaccines, many challenges are likely to slow down vaccine rollout. The lack of coordination among local organizations, inadequate monitoring and control of vaccine temperatures, logistical problems, inefficiency in maintaining vaccination target groups, miscommunication among the communities, weak health systems, weak political commitment, poor law enforcement, and vaccine hesitancy might all contribute to low vaccine coverage in LMICs [4]. Apart from operational challenges, vaccine hesitancy is a serious problem that could be caused by fear of side effects, lack of information and misinformation about vaccination, and distrust of government and pharmaceutical companies [5].

In January 2021, Egypt began vaccination against COVID-19 following a global strategy to prioritize older people after healthcare workers. Later on, the vaccine was available for free for all citizens who reserved shots on MoHP website application. Since COVID-19 vaccines were approved for use, MoHP started to collect administrative data on vaccination through the COVID-19 vaccination information system and the national disease surveillance (NEDSS). This data is used to assess vaccination coverage of COVID-19 at the national level, by governorate, and by selected sociodemographic characteristics. It is also used to calculate the quantities of vaccines used and required to achieve the vaccination targets in Egypt. MoHP has implemented this nationally representative survey to supplement the data on vaccine doses administered with information concerning public opinion towards COVID-19 vaccination and factors related to vaccine confidence.

All measures have been taken by Egypt to ensure vaccination is affordable, accessible, and attractive to the community. However, vaccine hesitancy among Egyptians is still an issue. Many families still had vaccine hesitancy for themselves and their children especially in remote areas and in children [6]. A study conducted by Cairo AUC recommended utilizing the “bottom/up” and “top/down approach” and mobilizing all relevant stakeholders in a campaign to raise awareness, dispel misconceptions and information about the vaccines, and build an open channel with the community [6]. With this in mind, Egypt MoHP, has recently started a knock-doors campaign led by community leaders inspecting the village’s houses spreading awareness on the vaccine, encouraging eligible family members to take the vaccine, and addressing rumors and misconceptions.

In Egypt, vaccination coverage and population perception about COVID-19 vaccines were studied through online surveys. Studies reported different acceptance rates among the Egyptian population ranging from 31.1 to 71.1% [7–9]. COVID-19 vaccine refusal was attributed to several factors in these studies, including doubt about vaccine effectiveness, lack of trust due to rapid vaccine production, fear of vaccines, lack of information, and fear of vaccine side effects. These results, however, lack representativeness due to the inappropriate survey designs.

Surveys are crucial for providing accurate estimates of vaccine coverage and understanding public opinion towards vaccination. However, survey results accuracy depends on maintaining statistical representativeness of their target populations by minimizing biases [10]. There is a need to reach reliable estimates of COVID-19 vaccine coverage and evaluate the efforts made to enhance vaccine uptake in Egypt. This nationwide community-based survey conducted by MoHP in March–May 2022, to better estimate COVID-19 vaccination coverage and understand people's perception of vaccination, in order to increase vaccination uptake and confidence among the Egyptian population and prioritize intervention strategies.

## Methods

### Study design

A nationally representative cross-sectional population-based household survey was implemented. Survey was conducted in two phases: the first phase was conducted at nine governorates using a multistage random sampling technique. In the second phase the survey was extended to cover the remaining 18 governorates using the same methodology.

### Sample size

A sample size of 6,000 subjects was calculated in the first nine governorates using epi info7 based on a full vaccination rate of 30% as estimated by the administrative data at the time of survey, with design effect of 2% and refusal rate of 10%. The survey was expanded to the remaining 18 governorates by targeting an additional 12,000 subjects, dividing them into 450 clusters of 20 households each. Number of study participants was distributed in proportion to each governorate’s population.

### Study setting

The study was conducted in all the Egyptian 27 governorates from March–May 2022. At each governorate, two districts with the highest and lowest rates of vaccination were selected using the administrative data. At each district, a number of rural and urban health offices were selected randomly to obtain the required clusters in proportion to the main occupation of the district population.

### Participants

Individuals living in the study area for the past six months, above 18 years of age, and present at the time of interview were eligible for the study regardless of their previous vaccination status. Subjects who consented verbally to participation after being informed of the study objectives and procedures were included. Children and adolescents under 18 were excluded from the study because vaccination for children under 18 have recently been approved in Egypt.

### Case definitions

The study subjects were categorized into the following categories based on the information in the vaccination cards.

### Vaccinated individuals

 Persons who received at least one dose of the vaccine.

### Partially vaccinated

Persons who did not receive the second dose of the two-doses COVID-19 vaccines.

### Fully vaccinated

Persons who have obtained the second dose of the two-dose vaccine or the first dose of the one-dose vaccine since ≥ 14 days prior to the survey date, without receiving a booster dose.

Receiver of booster doses: individuals who have already been fully vaccinated but have received a booster dose.

### Unvaccinated

 are those who did not receive any dose of the COVID-19 vaccine.

Vaccine breakthrough infection: individuals who were previously infected with COVID-19, as diagnosed by a physician or confirmed by RT-PCR after being fully vaccinated.

### Survey teams

Teams consisted of local community health workers from the selected districts, data collectors and field investigators under the supervision of MoHP epidemiologists. Pre-implementation training was provided by MoHP central-level epidemiologists who trained the local teams on survey methodology and roles. All questions were discussed in depth with the field teams during the training, and their comments were taken into account.

### Data collection

We used a semi-structured questionnaire adapted from Statistics Canada's COVID-19 vaccination coverage survey-Cycle2 [11].

The questionnaire was piloted in an unselected district to test its clarity and ease of use without including the data and was revised according to the pilot testing results and prior to actual data collection. Eligible subjects were interviewed face-to-face and data was entered on-site using Epi InfoTM mobile application by survey teams.

### Variables

The variables included participants' demographics, vaccination information including the number of vaccine doses received, dates, and vaccine types from the vaccination card, and history of COVID-19 infection and adverse events experienced after COVID-19 vaccines. The reasons for vaccine refusal included concerns about side effects, belief that vaccines are unbeneficial, difficulty registering, the notion that vaccines are unsafe during pregnancy and lactation, hearing that a COVID-19 vaccine can cause death, believing vaccines are unsafe for chronic disease patients, believing that a COVID-19 vaccine is not necessary, and being infected with COVID-19. Reasons for incomplete vaccination schedules include crowding, lack of organization, registration issues, and inaccessible vaccination centers and vaccine unavailability. Respondents were asked about their experiences and complaints with the vaccination centers during the client satisfaction survey e.g., crowds, disorganization, complicated registration process, inaccessibility, and whether or not they would recommend vaccination to friends and family.

### Bias

To avoid bias in data collection, the data collection tool was revised by MoHP senior epidemiologists and piloted before use in the survey. Data was entered on-site using an online application to obtain quality data. In the phase of data analysis, Multivariate logistic regression was used to exclude confounders linked to low vaccination uptake.

### Data analysis

Vaccination coverage rates were calculated by dividing the number of vaccinated and fully vaccinated subjects by the total number of participants. Vaccination coverage rates were compared by different demographic characteristics to identify the groups with low vaccine uptake. In order to identify the risk factors for low vaccine uptake, bivariate and multivariate analyses were conducted using vaccination status as the outcome variable, with Odds ratio, 95% confidence intervals, and a *p* value of 0.05. Vaccine breakthrough rates were calculated among vaccinated individuals to determine the appropriate vaccines to recommend for improving vaccine coverage.

The client satisfaction survey and reasons for refusal of COVID-19 vaccines were described using the opinions of those who verbally indicated receiving or not receiving vaccines to describe the level of satisfaction and reasons for refusal of vaccination uptake and identify gaps in the vaccination process.

### Ethical considerations

The study was approved by the Egyptian Ministry of Health and Population (MoHP) Research Ethics Committee (IRB).

## Results

### Demographics of participants

**Fig. 1:**
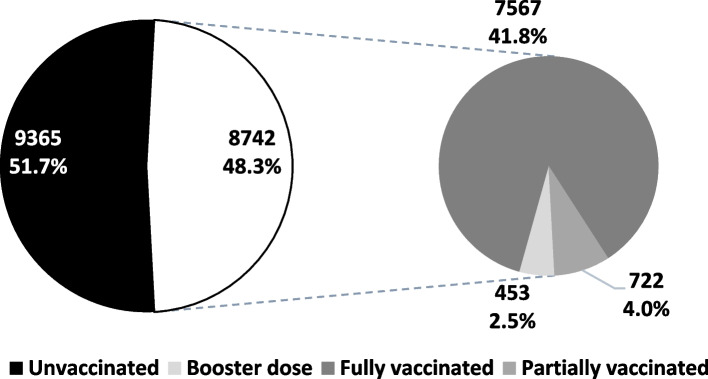
National survey estimates of vaccination rates

A total of 18,107 out of 18,462 selected subjects agreed to and completed the interview, representing a 98.1% response rate. Responders’ mean age was 42 ± 16 and 58.8% were females. Of them, 8,742 (48.3%) had at least one dose of the COVID-19 vaccine including 7,567 (41.8%) fully vaccinated, 722 (4.0%) partially vaccinated and 453 (2.5%) received at least one booster dose (Fig. 1).

Lower Vaccination rates were found among the age groups (18–29) and (30–49) years (39.6 and 46.8%), residents of urban and frontier governorates (39.0 and 44.5%), and females (47.7%) than other groups respectively (Table 1). Similarly, rate of full vaccination was lower among the age groups (18–29) and (30–49) years (34.3% and 42.7%), residents of urban and frontier governorates (36.1% and 41.4%), and females (43.4%) than other groups respectively. Whereas rates of vaccination and full vaccination were higher among the age group ≥ 65 years (55.7 and 53.0%), Lower Egypt residents (51.0 and 46.3%), and males (49.2 and 45.5%) than other groups respectively (Table 1).

**Table 1:** Rate of COVID-19 vaccination and full vaccination coverage by demographic characteristics of survey subjects, national vaccine coverage survey, March–May 2022

	**Total subjects** **(*n*=18,107)**	**Completed vaccination schedule**^**a**^** (*n*=8,020)**		**COVID-19 vaccinated**^**b**^** (n=8, 742)**	
		**Number**	**Row percent**	**Number**	**Row percent**
**Age group**					
18–29	3,756	1288	34.3	1,486	39.6
30–49	7,634	3259	42.7	3,574	46.8
50–64	4,647	2376	51.0	2,528	54.4
≥ 65	2070	1097	53.0	1,154	55.7
**Gender**					
Female	10,637	4621	43.4	5,070	47.7
Male	7,468	3399	45.5	3,671	49.2
**Governorate of residence**					
Urban	2,547	919	36.1	993	39.0
Frontier	818	339	41.4	364	44.5
Upper Egypt	6,859	3116	45.4	3,366	49.1
Lower Egypt	7,883	3646	46.3	4,019	51.0

^a^Includes fully vaccinated and booster dose receivers

^b^Includes partially vaccinated, fully vaccinated and booster dose receivers

Comparing demographic characteristics of vaccinated to unvaccinated individuals, it was found that the unvaccinated were significantly younger (mean age in years = 42.0 ± 15.5 vs 45.8 ± 15.5). Compared to the vaccinated group, risk of un-vaccination was higher among age groups (18–29) and (30–49) years (24.2vs 17.0% and 43.4 vs 40.9%), big families (mean number of family members (5.7 ± 3.6 vs 5.5 ± 3.3), primary and secondary educated (13.5 vs 12.1% and 37.5 vs 33.9%), housewives (49.1 vs 46.0%), self-employed (9.9 vs 8.3%), residents of urban and frontier governorates (16.6 vs 11.4% and 4.8 vs 4.2%) and married subjects (79.5 vs 77.5%) (Table 2). Multivariate analysis indicated that age 18–39 years, residents of urban and frontier governorates, housewives, married individuals and those who had below university education are significantly associated with un-vaccination status (Table 3).

**Table 2:** Comparison of demographic characteristics between study subject who had at least one dose of COVID-19 vaccination and those who never had COVID-19 vaccine

Characteristics	Total participants (*n =* 18,107)		COVID-19 vaccinated (*n =* 8,742)		COVID-19 unvaccinated (*n =* 9,365)		OR	95% CI	*P* value
	**Number**	**Percent**	**Number**	**Percent**	**Number**	**Percent**			
Mean age in years ± SD	43.8 ± 15.6		45.8 ± 15.5		42.0 ± 15.5				< 0.001
**Age groups (years)**									
18–29	3,756	20.7	1,486	17.0	2,270	24.2	0.64	0.60–0.69	< 0.001
30–49	7,634	42.2	3,574	40.9	4,060	43.4	0.90	0.85–0.96	< 0.001
50–64	4,647	25.7	2,528	28.9	2,119	22.6	1.39	1.30–1.49	< 0.001
≥ 65	2,070	11.4	1,154	13.2	916	9.8	1.40	1.28–1.54	< 0.001
**Gender**									
Males	7,468	41.2	3,671	42.0	3,797	40.5	1.06	1.00–1.13	0.050
Females	10,637	58.8	5,070	58.0	5,567	59.4	0.97	0.94–1.00	0.050
Mean number of family members ± SD	5.6 ± 3.4		5.5 ± 3.3		5.7 ± 3.6				< 0.001
**Education**									
Illiterate	5,961	32.9	3,105	35.5	2,856	30.5	1.26	1.18–1.34	< 0.001
Primary	2,318	12.8	1,056	12.1	1,262	13.5	0.88	0.81–0.96	0.005
Secondary	6,476	35.8	2,962	33.9	3,514	37.5	0.85	0.80–0.91	< 0.000
Higher education	3,350	18.5	1,618	18.5	1,732	18.5	1.00	0.93–1.08	0.995
**Occupation**									
Houswife	8612	47.6%	4018	46.0%	4594	49.1%	0.88	0.83–0.94	< 0.001
Unemployed	2343	12.9%	1275	14.6%	1068	11.4%	1.15	1.10–1.20	< 0.001
Employee	2126	11.7%	1047	12.0%	1079	11.5%	1.02	0.98–1.07	0.354
Worker	1883	10.4%	879	10.1%	1004	10.7%	0.96	0.92–1.01	0.149
Self-employed	1649	9.1%	725	8.3%	924	9.9%	0.90	0.85–0.96	< 0.001
Student	814	4.5%	447	5.1%	367	3.9%	1.14	1.07–1.22	< 0.001
Healthcare	360	2.0%	180	2.1%	180	1.9%	1.04	0.93–1.15	0.544
Teacher	320	1.8%	171	2.0%	149	1.6%	1.11	1.00–1.23	0.071
**Region**									
Urban governorates	2547	14.1	993	11.4	1554	16.6	0.64	0.59–0.70	< 0.001
Frontier governorates	818	4.5	364	4.2	454	4.8	0.85	0.74–0.98	0.029
Upper Egypt	6,859	37.9	3366	38.5	3493	37.3	1.05	0.99–1.12	0.097
Lower Egypt	7,883	43.5	4019	46.0	3864	41.3	1.21	1.14–1.28	< 0.001
**Marital status**									
Married	14,217	78.5	6772	77.5	7445	79.5	0.89	0.83–0.95	< 0.001
Single	2,262	12.5	1093	12.5	1169	12.5	1.00	0.92–1.10	0.985
Widow/divorced	1,626	9.0	876	10.0	750	8.0	1.28	1.16–1.42	< 0.001

**Table 3:** Risk factors associated with low vaccine uptake by multivariate analysis

Risk factor	OR	95% CI	*P*-value
Age 18–29	1.99	1.82–2.17	< 0.001
Urban governorate residence	1.63	1.49–1.77	< 0.001
Housewife	1.33	1.21–1.40	< 0.001
Married	1.29	1.19–1.40	< 0.001
Age 30–39	1.28	1.19–1.37	< 0.001
Self employed	1.22	1.10–1.37	< 0.001
Frontier governorate residence	1.22	1.05–1.40	0.008
Secondary education	1.11	1.04–1.19	0.002
Primary education	1.10	1.01–1.22	0.033

Of the 8,742 subjects who provided the vaccination cards, 3,530 (40.4%) had inactivated vaccine type, 3,015 (34.5%) viral vector, 2,142 (24.5%) mRNA and 55 (0.6%) had more than one type.

Percentage of the full vaccination was higher and adverse events were lower among the inactivated type receivers compared to the viral vector and mRNA types (94.7 vs 90.1 vs 88.8%) and (32.0 vs 46.1 vs 50.1%) respectively. Whereas vaccine breakthrough infection was higher among the inactivated vaccine compared to viral vector and mRNA types (1.6 vs 1.4 vs 0.9%) respectively (Table 4).

**Table 4:** Level of vaccination, vaccine breakthrough infection rate, and adverse events by vaccine type, national vaccine coverage survey, Egypt, March–May 2022

Level of vaccination	Total vaccinated (*n =* 8,742)		Inactivated (*n =* 3,530)		Viral vector (*n =* 3,015)		mRNA (2,142)		Heterologous (*n =* 55)	
	**Number**	**Percent**	**Number**	**Percent**	**Number**	**Percent**	**Number**	**Percent**	**Number**	**Percent**
Full vaccination	7567	86.6%	3122	88.4%	2576	85.4%	1854	86.6%	15	27.3%
Booster	453	5.2%	224	6.3%	142	4.7%	47	2.2%	40	72.7%
Partial vaccination	722	8.3%	184	5.2%	297	9.9%	241	11.3%	0	0.0%
Vaccine breakthrough	117	1.3%	55	1.6%	42	1.4%	19	0.9%	0	0.0%
Adverse events	3618	41.4%	1129	32.0%	1391	46.1%	1074	50.1%	24	43.6%

Among 3,996 who mentioned not having the vaccination, vaccine hesitancy was primarily due to fear of vaccine adverse events (41.0%), mistrust of vaccine benefits (24.0%), difficulties in registration (19.4%), concerns over safety during pregnancy and lactation (16.2%), and safety for chronic diseases patients (11.7%). While among 1,166 subjects who verbally reported partial vaccination, 6.0% complained of crowdedness and disorganization at vaccine centers, and 4.4% experienced registration difficulties (Table 5).

**Table 5:** Reasons for non-vaccination against COVID-19 reported by the unvaccinated and partially vaccinated participants

	No	Percent
**Reasons for vaccination refusal among unvaccinated individuals (*n =* 3,996)**		
I am concerned about possible side effects of a COVID-19 vaccine	1,637	41.0%
I do not think vaccines are beneficial	959	24.0%
I faced difficulty in registration	776	19.4%
I think vaccines unsafe during pregnancy and lactation	649	16.2%
I think vaccines unsafe for chronic disease patients	466	11.7%
I do not believe I need a COVID-19 vaccine	162	4.1%
I heard that COVID-19 vaccines can lead to death	47	1.2%
I already had COVID-19	34	0.9%
**Reasons for incomplete vaccination schedule among partially vaccinated individuals (*n =* 1,166)**		
Intend to complete the vaccination	650	55.7%
Crowded disorganized vaccination center	70	6.0%
Problem with registration	51	4.4%
Vaccine unavailable	22	1.9%
Inaccessible center	20	1.7%

**Fig. 2:**
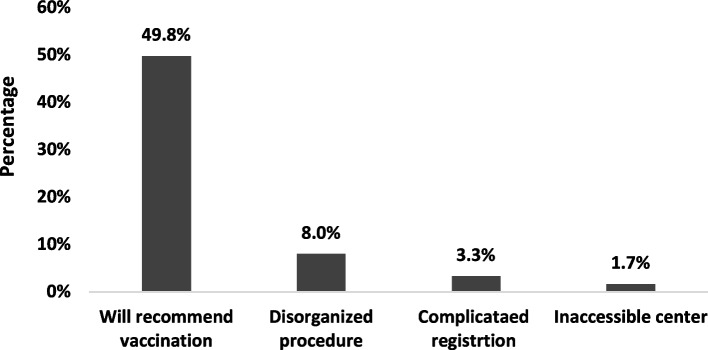
Experience of vaccinated subjects with the vaccination process (*n =* 14,111)

The client satisfaction survey among 14,111 subjects who mentioned having at least one dose of COVID-19 vaccine indicated that almost half of them were satisfied with the service and would recommend vaccination to others. Whereas 8.0% complained of disorganization and crowding at vaccination centers, 3.3% encountered difficulty registering using the online application, and 1.7% found difficulty in reaching the vaccination center (Fig. 2).

## Discussion

COVID-19 is likely to remain in the global public health landscape in the near future. Given the increasing evidence that vaccines are effective in preventing fatalities, hospitalizations, and severe cases, it is imperative to assess the risk of low vaccine uptake in different communities. Furthermore, it is important to understand how people perceive COVID-19 vaccinations in order to prioritize intervention strategies for improving vaccination uptake and increasing confidence in the vaccine [12]. Almost all of the studies conducted in Egypt to estimate COVID-19 vaccination coverage and assess vaccine hesitancy targeted specific groups or only included those who could read and have access to the internet [7–9]. This is the first and largest household survey conducted in Egypt to estimate COVID-19 vaccination coverage and identify causes of low vaccine uptake among the Egyptian population.

### Vaccination coverage survey

COVID-19 is approaching endemicity, and national surveys are essential to monitoring vaccination coverage in order to identify gaps in vaccine coverage and provide useful information regarding vaccine uptake by different sociodemographic characteristics and reasons for low vaccine uptake. This should help develop effective intervention strategies to increase confidence in vaccines and promote vaccine uptake.

The survey indicated that more than half of the Egyptian population ≥ 18 years are unvaccinated. As approximately 40% of Egyptians are under 18 years of age, this will even result in lower vaccination coverage for the whole population since vaccination rates are very low in this age group. More efforts are needed to reach the WHO target of 70% of the whole population to contain the pandemic [13]. Currently, MoHP is conducting a knock-door vaccination campaign based on the results of this survey to encourage vaccine uptake all over the country.

### Sociodemographic determinants of low vaccine uptake

According to our study, vaccine coverage was lower among young adults, female housewives, married individuals, low-educated, large families, and those living in urban and frontier areas. Previous studies reported greater COVID-19 vaccine hesitancy among younger ages [14–16]. Other studies have shown that young people believe that they are not at higher risk for COVID-19 infection, that the vaccine is not necessary, or that COVID-19 is not a serious disease in accordance with our study [14, 15].

Results of the studies investigating COVID-19 vaccine hesitancy among the rural and urban populations are controversial, prior studies showed greater hesitancy among residents of rural areas, while others did not find a difference between rural or urban area residents in vaccine hesitancy [14, 15, 17]. This study found lower COVID-19 vaccine uptake among residents of urban governorates. A number of factors may contribute to this, including mistrust of COVID-19 vaccines caused by the use of social media as a source of information that is untrustworthy, difficulties getting vaccinations due to overcrowded vaccination centers, and difficulties implementing house-to-house vaccination campaigns in urban areas. Previous studies reported higher rates of vaccine hesitancy among females whereas others did not find a significant difference between males and females regarding vaccine uptake [15–17]. This study did not find a difference between females and males in vaccine uptake, however, we found that 16.2% of the unvaccinated are having concerns about the safety of COVID-19 vaccines for pregnant and lactating mothers. Recent studies proved that the COVID-19 vaccinations are safe and efficient in pregnant and lactating women and also conferred benefits to newborns [18]. A discussion has taken place among obstetricians on social media in Egypt regarding the safety of COVID-19 vaccinations for pregnant women, women planning to become pregnant, and nursing women. Obstetricians should monitor the most recent research on this topic.

Unemployment and low-income families were found to be associated with vaccine hesitancy in other studies [19, 20]. In contrast, higher vaccination rates were found in this study among the unemployed, which could be related to retirement status and old age, as the elderly have a higher rate of vaccination. A lower vaccination rate was observed among housewives with large families, probably because they had lower incomes. Self-employed people were found to have a lower vaccination rate, while students and teachers have significantly higher vaccination coverage. These findings could be explained by the vaccination requirements for university students, teachers, and government employees. Additionally, the Egyptian government has mandated vaccinations for attendees in all government institutions, however, this is logistically challenging. The current knock-door outreach campaign requiring no public attendance could promote vaccine uptake among housewives and self-employed individuals.

The level of education was found to be associated with vaccine uptake in this study. Studies found that individuals with lower education levels are having lower vaccine uptake than individuals with a university degree [19, 20]. The data in our study show a mixed picture. In accordance with other studies, we found a low level of uptake among the below university-educated, however, significantly higher vaccine uptake was found among illiterates. This could be due to the older age of illiterates in Egypt and the successful communication strategy for old ages [21].

This study found that married individuals had lower vaccination uptake than divorced/widowed individuals. This could indicate that those who live alone are more concerned about COVID-19 infection and more eager to get the vaccine.

According to our study, > 90% of the vaccinated individuals have completed their vaccination schedule, a small percentage are partially vaccinated, and almost half of them have future appointments. Our study supports targeting groups who refuse vaccination rather than those who are partially.

A higher percentage of full vaccination was observed in this study among inactivated vaccine recipients than among other vaccine types, possibly because inactivated vaccine recipients experienced fewer adverse events [22]. However, this study identified higher rates of vaccine breakthrough infections among inactivated type receivers, with no breakthrough infections were identified among receivers of heterologous vaccination regimen. In Egypt, there are multiple types of COVID-19 vaccines available, so the vaccine used to improve coverage should be selected after careful consideration and comparison of the advantages and disadvantages of different types with considering its effectiveness against prevailing mutants.

### Behavioral determinants of low vaccine uptake

The main reason for COVID-19 vaccines low uptake found in this study was the caution and safety concerns about side effects. The rapid development of COVID-19 vaccines and the accelerated approval process associated with these vaccines might be contributing factors to the distrust in vaccine safety. In addition to the ongoing debate about COVID-19 variants and whether vaccines work against circulating new variants, the vaccination development process might take a decade or more [16].

The lack of confidence in vaccine effectiveness was the second cause of un-vaccination in Egypt. It has been found that individuals who are less afraid of COVID-19 infection are more likely to report vaccine hesitancy [12, 14]. The misperception of the low risk of getting COVID-19, especially at young ages noted in our study and by others could be related to the relatively low incidence and mortality rates associated with the pandemic reported [14–17, 23]. The general public should be informed COVID-19 situation in a balanced manner while maintaining full transparency and preventing panic among the public [24]. In addition, it was found that trusted public sector officials play a key role in improving vaccination rates [23].

Misinformation regarding safety of the vaccine for pregnant and lactating females identified in this survey as a cause of low vaccine uptake was also reported as a cause of vaccine hesitancy in previous studies [16, 18]. A study reported that 7% of women were unwilling to receive the vaccination due to pregnancy or lactation [16]. COVID-19 vaccination was prioritized for patients with chronic diseases due to the risk of COVID-19 morbidity and mortality associated with these conditions [25]. A study conducted in the United States found that chronic disease status is significantly associated with willingness to be vaccinated [26]. In contrast with this, our study found that concern about the safety of the vaccine for patients with chronic diseases was one of the causes of low vaccine uptake. To promote vaccine uptake, a communication campaign that addresses misinformation regarding the safety of COVID-19 vaccines for pregnant, lactating, and chronic disease patients should be implemented.

In Egypt, a hotline has been assigned to direct the public on how to register for vaccination appointments using the web-based application, but difficulties have been reported. To improve vaccine registration and uptake, it is imperative to assess the gaps in using the application, as well as the performance of the hotline and mobile teams.

The results of the client satisfaction survey indicated that most of those who were vaccinated were satisfied with the service and reported that they would recommend the vaccine to others. However, complaints were made about crowded conditions and disorganization during vaccination, particularly in urban governorates, the lack of certain vaccines, and the assignment to vaccination centers far from their homes. A system of complaints could help identify and solve the operational problems in the vaccination process.

## Study limitations

The study has many limitations, first, the study results were unweighted based on Egypt population, and hence rates could be overestimated. Second is the large sample size of the survey data, which may increase the significance of the results, and third vaccination rates calculated for each demographic group were unweighted. In addition, district selection in each governorate was not random, but the two districts were selected based on vaccination coverage and rural-to-urban areas to represent all opinions. The study was conducted in 2 phases, with the second phase comprising more populations, however both phases were completed within a three months period. We think that the sample is representative of Egypt’s population as the sample size is adequate and the sample was obtained from all the 27 Egyptian governorates in accordance with the population size.

## Conclusions

The national survey identified low vaccination coverage rates in Egypt compared to the 70% WHO target, all efforts should be made to promote vaccine uptake to achieve the target necessary to drive the pandemic to an end. The study has pointed out to certain groups including younger ages, housewives, self-employed, urban and frontier governorates residents, and low educated that should be targeted for an intervention to maximize vaccine uptake through enhanced public health messaging. Communications programs that deal with the concerns of vaccine side effects, vaccine mistrust, and misinformation about vaccine safety are required to effectively promote vaccine uptake. The door to door “knock-doors” vaccination campaign implemented recently by MoHP using these study recommendations could improve vaccine coverage through treating difficulty in registration, crowdedness and disorganization at vaccination centers. Findings from the survey could contribute significantly to vaccination promotion by guiding decision-making efforts on the risky groups for preventing vaccine hesitancy.

## Data Availability

The datasets generated and/or analyzed during the current study are not publicly available due to privacy restrictions but are available from the Egyptian Ministry of Health and Population on reasonable request.
